# On the epidemiology of influenza

**DOI:** 10.1186/1743-422X-5-29

**Published:** 2008-02-25

**Authors:** John J Cannell, Michael Zasloff, Cedric F Garland, Robert Scragg, Edward Giovannucci

**Affiliations:** 1 Department of Psychiatry, Atascadero State Hospital, 10333 El Camino Real, Atascadero, CA 93423, USA; 2 Departments of Surgery and Pediatrics, Georgetown University, Washington, D.C., USA; 3 Department of Family and Preventive Medicine, University of California San Diego, La Jolla, CA, USA; 4 Department of Epidemiology and Biostatistics, University of Auckland, Auckland, New Zealand; 5 Departments of Nutrition and Epidemiology, Harvard School of Public Health, Boston, MA, USA

## Abstract

The epidemiology of influenza swarms with incongruities, incongruities exhaustively detailed by the late British epidemiologist, Edgar Hope-Simpson. He was the first to propose a parsimonious theory explaining why influenza is, as Gregg said, "seemingly unmindful of traditional infectious disease behavioral patterns." Recent discoveries indicate vitamin D upregulates the endogenous antibiotics of innate immunity and suggest that the incongruities explored by Hope-Simpson may be secondary to the epidemiology of vitamin D deficiency. We identify – and attempt to explain – nine influenza conundrums: (1) Why is influenza both seasonal and ubiquitous and where is the virus between epidemics? (2) Why are the epidemics so explosive? (3) Why do they end so abruptly? (4) What explains the frequent coincidental timing of epidemics in countries of similar latitude? (5) Why is the serial interval obscure? (6) Why is the secondary attack rate so low? (7) Why did epidemics in previous ages spread so rapidly, despite the lack of modern transport? (8) Why does experimental inoculation of seronegative humans fail to cause illness in all the volunteers? (9) Why has influenza mortality of the aged not declined as their vaccination rates increased? We review recent discoveries about vitamin D's effects on innate immunity, human studies attempting sick-to-well transmission, naturalistic reports of human transmission, studies of serial interval, secondary attack rates, and relevant animal studies. We hypothesize that two factors explain the nine conundrums: vitamin D's seasonal and population effects on innate immunity, and the presence of a subpopulation of "good infectors." If true, our revision of Edgar Hope-Simpson's theory has profound implications for the prevention of influenza.

## Introduction

It is useful, at times, to question our assumptions. Arguably, the most universally accepted assumption about influenza is that it is a highly infectious virus spread by the sick. Edgar Hope-Simpson not only questioned that assumption, he went much further. Realizing that solar radiation has profound effects on influenza, he added an unidentified "seasonal stimulus" to the heart of his radical epidemiological model [1]. Unfortunately, the mechanism of action of the "seasonal stimulus" eluded him in life and his theory languished. Nevertheless, he parsimoniously used latent asymptomatic infectors and an unidentified "season stimulus" to fully or partially explain seven epidemiological conundrums [2].

1. Why is influenza both seasonal and ubiquitous and where is the virus between epidemics?

2. Why are the epidemics so explosive?

3. Why do epidemics end so abruptly?

4. What explains the frequent coincidental timing of epidemics in countries of similar latitudes?

5. Why is the serial interval obscure?

6. Why is the secondary attack rate so low?

7. Why did epidemics in previous ages spread so rapidly, despite the lack of modern transport?

An eighth conundrum – one not addressed by Hope-Simpson – is the surprising percentage of seronegative volunteers who either escape infection or develop only minor illness after being experimentally inoculated with a novel influenza virus. The percentage of subjects sickened by iatrogenic aerosol inoculation of influenza virus is less than 50% [3], although such experiments depend on the dose of virus used. Only three of eight subjects without pre-existing antibodies developed illness after aerosol inhalation of A_2_/Bethesda/10/63 [4]. Intranasal administration of various wild viruses to sero-negative volunteers only resulted in constitutional symptoms 60% of the time; inoculation with Fort Dix Swine virus (H_1_N_1_) – a virus thought to be similar to the 1918 virus – in six sero-negative volunteers failed to produce any serious illness, with one volunteer suffering moderate illness, three mild, one very mild, and one no illness at all [5]. Similar studies by Beare *et al *on other H_1_N_1 _viruses found 46 of 55 directly inoculated volunteers failed to develop constitutional symptoms [6]. If influenza is highly infectious, why doesn't direct inoculation of a novel virus cause universal illness in seronegative volunteers?

A ninth conundrum evident only recently is that epidemiological studies question vaccine effectiveness, contrary to randomized controlled trials, which show vaccines to be effective. For example, influenza mortality and hospitalization rates for older Americans significantly increased in the 80's and 90's, during the same time that influenza vaccination rates for elderly Americans dramatically increased [7,8]. Even when aging of the population is accounted for, death rates of the most immunized age group did not decline [9]. Rizzo *et al *studying Italian elderly, concluded, "We found no evidence of reduction in influenza-related mortality in the last 15 years, despite the concomitant increase of influenza vaccination coverage from ~10% to ~60%" [10]. Given that influenza vaccinations increase adaptive immunity, why don't epidemiological studies show increasing vaccination rates are translating into decreasing illness?

After confronting influenza's conundrums, Hope-Simpson concluded that the epidemiology of influenza was not consistent with a highly infectious disease sustained by an endless chain of sick-to-well transmissions [2]. Two of the three most recent reviews about the epidemiology of influenza state it is "generally accepted" that influenza is highly infectious and repeatedly transmitted from the sick to the well, but none give references documenting such transmission [11-13]. Gregg, in an earlier review, also reiterated this "generally accepted" theory but warned:

"Some fundamental aspects of the epidemiology of influenza remain obscure and controversial. Such broad questions as what specific forces direct the appearance and disappearance of epidemics still challenge virologists and epidemiologists alike. Moreover, at the most basic community, school, or family levels of observation, even the simple dynamics of virus introduction, appearance, dissemination, and particularly transmission vary from epidemic to epidemic, locale to locale, seemingly unmindful of traditional infectious disease behavioral patterns." [14] (p. 46)

Questioning a generally accepted assumption means asking anew, "What does the evidence actually show? Thus, we asked, are there any controlled human studies that attempted sick-to-well influenza transmission? Do naturalistic studies of outbreaks in confined spaces prove sick-to-well transmission or are they compatible with another mode of dissemination? Is there an easily measurable serial interval (the median time between the index case and the secondary cases), so crucial to establishing sick-to-well transmission? Are measured secondary attack rates in families (the percentage of family members sickened after a primary case) suggestive of a highly infectious virus? What do animal models of influenza tell us?

Do current theories explain the explosive onset and then abrupt disappearance of epidemics, epidemics that cease despite a wealth of potential victims lacking adaptive immunity [15]? Why have epidemic patterns in Great Britain not altered in four centuries, centuries that have seen great increases in the speed of human transport [16]? If each successive epidemic increases herd immunity and children born since the last epidemic are non-immune, why doesn't the average age of persons infected in successive epidemics become progressively lower[17]? Why did the peak of 25 consecutive epidemics in France and the USA occur within a mean of four days of each other [18]?

Review of Jordan's sobering monograph of the 1918 pandemic leaves little room to doubt that close human interaction propagates influenza [19]. Furthermore, laboratory evidence leaves no doubt that droplets or aerosols can transmit influenza; droplets containing a high dose of virus, or aerosols containing a much lower dose, both can result in iatrogenic human infection [20].

Subjects that sicken do so two to four days after being iatrogenically infected; that is, the incubation period is about three days.* However, it is crucial to remember that the incubation period only tells us what the serial interval should be, not what it is. Furthermore, induction of human infection in the laboratory only tells us such infection is possible; it does not tell us who is infecting the well in nature*.

The obvious candidate is the sick. However, Edgar Hope-Simpson contended that the extant literature on serial interval, secondary attack rates, and other epidemiological aspects of influenza are not compatible with sick-to-well transmission as the usual mode of contagion. In his 1992 book, after considering all known epidemiological factors, he presented a comprehensive, parsimonious – and radically different – model for the transmission of influenza, one heavily dependent on a profound, even controlling, effect of solar radiation. Furthermore, while agreeing the sick could infect the well, Hope-Simpson's principal hypothesis was that epidemic influenza often propagates itself by a series of transmissions from a small number of highly infectious – but generally symptomless – latent carriers, briefly called into contagiousness by the "seasonal stimulus."

In contrast, Kilbourne's 1987 text – without mentioning serial interval or secondary attack rates in his chapter on epidemiology – concluded, "Any doubt about the communicability of influenza from those ill with the disease is dispelled by studies in crowded, confined, or isolated populations" [21]. (p. 269) As discussed below, the naturalistic studies Kilbourne refers to certainly indicate human interaction facilitates transmission of influenza. However, these naturalistic studies simply assume that the first person with identified illness is the index case. Obviously, A preceding B does not prove A causes B.

## Vitamin D, innate immunity, and influenza

**Figure 1:**
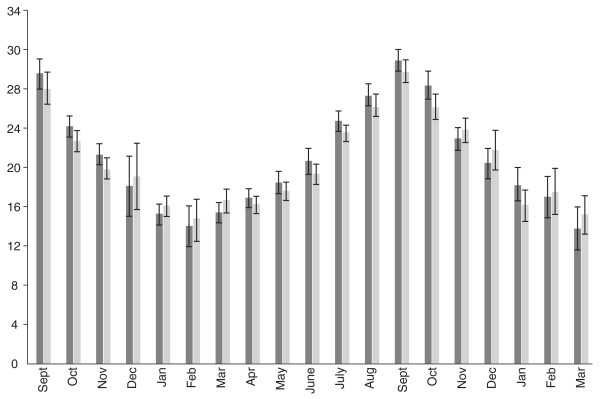
**Geometric mean monthly variations in serum 25-hydroxyvitamin D [25)OH)D] concentration in men (dark shade, n = 3723) and women (light shade, n = 3712) in a 1958 British birth cohort at age 45.** 25(OH)D levels are in ng/ml; to convert to nmol/L, multiply by 2.5. Adapted from: Hypponen E, Power C: Hypovitaminosis D in British adults at age 45 y: nationwide cohort study of dietary and lifestyle predictors. *Am J Clin Nutr *2007, **85**: 860–868. Reproduced with kind permission of the American Society for Nutrition.

Hope-Simpson's model theorized that an unidentified "seasonal stimulus," inextricably bound to solar radiation, substantially controlled the seasonality of influenza. Recent evidence suggests the "seasonal stimulus" may be seasonal impairments of the antimicrobial peptide (AMPs) systems crucial to innate immunity [22], impairments caused by dramatic seasonal fluctuations in 25-hydroxy-vitamin D [25(OH)D] levels [23]. (Figure 1) The evidence that vitamin D has profound effects on innate immunity is rapidly growing [24].

**Figure 2:**
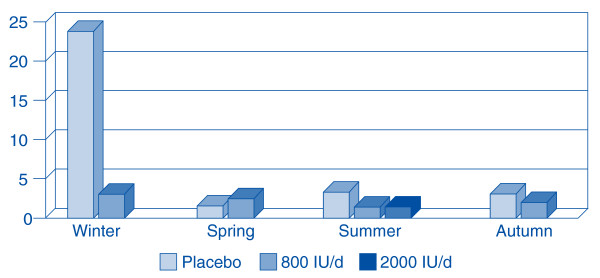
**Incidence of reported cold/influenza symptoms according to season.** The 104 subjects in the placebo group (light shade) reported cold and flu symptoms year around with the most symptoms in the winter. While on 800 IU per day (intermediate shade) the 104 test subjects were as likely to get sick in the summer as the winter. Only one of the 104 test subjects had cold/influenza symptoms during the final year of the trial, when they took 2,000 IU of vitamin D per day (dark shading). Adapted from: Aloia JF, Li-Ng M: Epidemic influenza and vitamin D. Epidemiol Infect 2007; 135: 1095–1096. (Reproduced with permission, Cambridge University Press).

In fact, Aloia and Li-Ng presented evidence of a dramatic vitamin D preventative effect from a randomized controlled trial (RCT) [25]. In a *post-hoc *analysis of the side effect questions of their original three-year RCT, they discovered 104 post-menopausal African American women given vitamin D were three times less likely to report cold and flu symptoms than 104 placebo controls. A low dose (800 IU/day) not only reduced reported incidence, it abolished the seasonality of reported colds and flu. A higher dose (2000 IU/day), given during the last year of their trial, virtually eradicated all reports of colds or flu. (Figure 2) Recent discoveries about vitamin D's mechanism of action in combating infections [26] led *Science News *to suggest that vitamin D is the "antibiotic vitamin" [27] due primarily to its robust effects on innate immunity.

Unlike adaptive immunity, innate immunity is that branch of host defense that is "hard-wired" to respond rapidly to microorganisms using genetically encoded effectors that are ready for activation by an antigen before the body has ever encountered that antigen. Activators include intact microbes, Pathogen Associated Molecular Patterns (PAMPS), and host cellular constituents released during tissue injury. Of the effectors, the best studied are the antimicrobial peptides (AMPs) [28].

Both epithelial tissues and phagocytic blood cells produce AMPs; they exhibit rapid and broad-spectrum antimicrobial activity against bacteria, fungi, and viruses [29]. In general, they act by rapidly and irreversibly damaging the lipoprotein membranes of microbial targets, including enveloped viruses, like influenza [30]. Other AMPs, such as human beta-defensin 3, inhibit influenza haemagglutinin A mediated fusion by binding to haemagglutinin A associated carbohydrates via a lectin-like interaction [31].

AMPs protect mucosal epithelial surfaces by creating a hostile antimicrobial shield. The epithelia secrete them constitutively into the thin layer of fluid that lies above the apical surface of the epithelium but below the viscous mucous layer. To effectively access the epithelium a microbe, such as influenza, must penetrate the mucous barrier and then survive damage inflicted by the AMPs present in the fluid that is in immediate contact with the epithelial surface. Should this constitutive barrier be breached, the binding of microbes to the epithelium and/or local tissue injury rapidly provokes the expression of high concentrations of specific inducible AMPs such as human beta-defensin 2 and cathelicidin, that provide a "back-up" antimicrobial shield. These inducible AMPs also act as chemo-attractants for macrophages and neutrophils that are present in the immediate vicinity of the site of the microbial breach [28-30]. In addition, cathelicidin plays a role in epithelial repair by triggering epithelial growth and angiogenesis [32].

The crucial role of vitamin D in the innate immune system was discovered only very recently [33,34]. Both epithelial cells and macrophages increase expression of the antimicrobial cathelicidin upon exposure to microbes, an expression that is dependent upon the presence of vitamin D. Pathogenic microbes, much like the commensals that inhabit the upper airway, stimulate the production of a hydroxylase that converts 25(OH)D to 1,25(OH)_2_D, a seco-steroid hormone. This in turn rapidly activates a suite of genes involved in defense [35].

In the macrophage, the presence of vitamin D also appears to suppress the pro-inflammatory cytokines, Interferon γ, TNFα, and IL12, and down regulate the cellular expression of several PAMP receptors. In the epidermis, vitamin D induces additional PAMP receptors, enabling keratinocytes to recognize and respond to microbes [36]. Thus, vitamin D appears to both enhance the local capacity of the epithelium to produce endogenous antibiotics and – at the same time – dampen certain arms of the adaptive immune response, especially those responsible for the signs and symptoms of acute inflammation, such as the cytokine storms operative when influenza kills quickly.

Of particular note is that not all animals appear to depend on vitamin D for their innate immune circuitry. The cathelicidin genes of mouse, rat, and dog, lack a vitamin D receptor-binding site, and do not require vitamin D for expression [34]. Therefore, one cannot extrapolate the role vitamin D plays in human infections from studies of such animals.

Plasma levels of vitamin 25(OH)D in African Americans, known to be lower than white skinned individuals, are inadequate to fully stimulate the vitamin D dependent antimicrobial circuits operative within the innate immune system. However, the addition of 25(OH)D restored the dependent circuits and greatly enhanced expression of AMPs [37]. High concentrations of melanin in dark-skinned individuals shield the keratinocytes from the ultraviolet radiation required to generate vitamin D in skin [38]. In addition, the production of vitamin D in skin diminishes with aging [39]. Therefore, relative – but easily correctable – deficiencies in innate immunity probably exist in many dark-skinned and aged individuals, especially during the winter.

Because humans obtain most vitamin D from sun exposure and not from diet, a varying percentage of the population is vitamin D deficient, at any time, during any season, at any latitude, although the percentage is higher in the winter, in the aged, in the obese, in the sun-deprived, in the dark-skinned, and in more poleward populations [40,41]. However, seasonal variation of vitamin D levels even occur around the equator [42] and widespread vitamin D deficiency can occur at equatorial latitudes [43], probably due to sun avoidance [44], rainy seasons [45], and air pollution [46]. For example, a study of Hong Kong infants showed about half had 25(OH)D levels less than 20 ng/ml in the winter [47]. Even in the summer, few of the infants had levels higher than 30 ng/ml, which many experts now think are below the lower limit of the optimal range [40,41,48,49]. As 25(OH)D levels affect innate immunity, then a varying percentage of most populations – even equatorial ones – will have impaired innate immunity at any given time, together with distinct seasonal variations in that percentage. The effects such impairments have on influenza transmission are unknown.

## Human studies attempting sick-to-well human transmission

In 2003, Bridges *et al *reviewed influenza transmission and found "no human experimental studies published in the English-language literature delineating person-to-person transmission of influenza. This stands in contrast to several elegant human studies of rhinovirus and RSV transmission ..." [50]. (p. 1097)

However, according to Jordan's frightening monograph on the 1918 pandemic, there were five attempts to demonstrate sick-to-well influenza transmission in the desperate days following the pandemic and all were "singularly fruitless" [19]. (p. 441) Jordan reports that all five studies failed to support sick-to-well transmission, in spite of having numerous acutely ill influenza patients, in various stages of their illness, carefully cough, spit, and breathe on a combined total of >150 well patients [51-55].

Rosenau's work was the largest of the studies, illustrative of the attempts, and remarkable for the courageousness of the volunteers [52]. In 1919 – in a series of experiments – he and six colleagues at the U.S. Public Health Service attempted to infect 100 "volunteers obtained from the Navy." He reports all volunteers were "of the most susceptible age," and none reported influenza symptoms in 1918. That is, "from the most careful histories that we could elicit, they gave no account of a febrile attack of any kind," during the previous year. The authors then selected influenza donors from patients in a "distinct focus or outbreak of influenza, sometimes an epidemic in a school with 100 cases, from which we would select typical cases, in order to prevent mistakes in diagnosis of influenza." Rosenau made every attempt to get donors who were early in their illness, "A few of the donors were in the first day of the disease. Others were in the second or third day of the disease."

"Then we proceeded to transfer the virus obtained from cases of the disease; that is, we collected the material and mucous secretions of the mouth and nose and bronchi from (19) cases of the disease and transferred this to our volunteers. We always obtained the material in the following way: The patients with fever, in bed, has a large, shallow, traylike arrangement before him or her, and we washed out one nostril with some sterile salt solution, using perhaps 5 c.c., which is allowed to run into this tray; and that nostril is blown vigorously into the tray. That is repeated with the other nostril. The patient then gargles the solution. Next we obtain some bronchial mucous through coughing, and then we swab the mucous surface of each nares and also the mucous membranes of the throat."

Then they mixed all the "stuff" together and sprayed 1 cc of the mixture in each of the nostrils of 10 volunteers, and "into the throat, while inspiring, and on the eye" and waited 10 days for the volunteers to fall ill. However, "none of them took sick in any way." Undaunted, Rosenau conducted another experiment in which ten acutely ill influenza patients coughed directly into the faces of each ten well volunteers. Again, "none of them took sick in any way."

Perhaps Rosenau's and similar experiments failed because all the well volunteers had contracted infections in 1918 and were immune from further infection. While possible, none of the volunteers reported symptoms in 1918, even a fever. Furthermore, adaptive immunity to influenza is relative to the immune response that infection generates and to the time since infection; it is seldom absolute and abiding.

Another explanation is that all of the influenza patients had passed their time of infectivity although Rosenau obtained donors in the first, second, or third day of their illnesses. As no laboratory confirmation was possible, perhaps the ill did not have influenza, but we doubt U.S. Public Health Service physicians had much trouble making accurate clinical diagnosis of influenza in 1919. Furthermore, all the donors were symptomatic; peak viral shedding occurs 24–72 hours after infection, and the amount of virus shed is associated with symptoms [56]. Perhaps peak viral shedding is not associated with peak infectivity. Perhaps – although Rosenau does not report the date or season of the experiments – all the naval volunteers had adequate innate immunity from sun exposure. Obviously, another explanation is that sick-to-well transmission is not the usual mode of contagion.

## Naturalistic reports of sick-to-well transmission

A number of naturalistic studies suggest influenza is transmitted from the sick to the well [57-59]. They all assume the first case was the index case. The best-known case is an airliner in Alaska, where an extensive outbreak of influenza occurred after an infected patient appeared among well, and the airliner subsequently malfunctioned, causing a four-hour delay in which passengers breathed re-circulated air [60].

Although her influenza culture was negative, the authors hypothesized their "index case" infected 37 well passengers within a mean of 38 hours after she boarded the plane. However, 30 other passengers boarded the Alaskan plane at the same time as the sick passenger, and other passengers were already onboard, any of whom could have been the common source. The airline study, like other naturalistic studies, is very suggestive of a common source and aerosol transmission, but offers no proof that the common source was the suspected index case, other than the logic that if A preceded B then A must have caused B.

Experts frequently cite an experience at an "irradiated" Livermore, California, VA hospital during the 1957–58 influenza epidemic as naturalistic evidence of sick-to-well aerosol influenza transmission. McLean (as part of a general discussion in a paper by Jordan) [61] reported an entire hospital building unit, housing approximately 150 patients with chronic pulmonary disease, was "totally radiated" in an attempt to reduce TB contagion through the air. There remained, nonradiated, another 250 control patients. He reported a two percent influenza attack rate for the "radiated patients" compared to a 19 percent attack rate for the "nonradiated patients." (p. 37).

However, Maclean's description of the Livermore hospital's irradiation procedures is inadequate to know if patients were being directly irradiated, thus triggering vitamin D production in their skin. However, careful inspection of another 1957 publication about a similarly irradiated Baltimore VA hospital – co-authored by McLean – is illuminating [62]. The Baltimore hospital wing apparently used a similar irradiation set-up with "standard ultraviolet light fixtures." (p. 421) Illustrations clearly show – despite text stating that only upper air was irradiated – that the rooms and hallways were all equipped with UV lights that either shone directly or indirectly on patients, apparently 24 hours per day, seven days a week (see pp. 422–423 for illustrations). If the irradiation processes were similar in Livermore and Baltimore hospitals, they would have significantly raised the 25(OH)D levels of the irradiated, and relatively influenza-free, patients.

Furthermore, if irradiation of the air destroyed viral aerosols and was responsible for the lower attack rate, such results should be reproducible. In a carefully controlled trial, Gelperin *et al *directly investigated the possibility of transmission of viral respiratory illness by aerosols [63]. For four months during the height of the flu season, the authors carefully irradiated only the upper air in half the classrooms in eight New Haven schools with ultraviolet light, and, unlike the Livermore VA hospital, the researchers took great care not to irradiate the students, either directly or indirectly. When they compared absenteeism in irradiated classrooms to non-irradiated control schools, they found no effect from upper air irradiation. Two other large field studies in schools likewise showed no effect from UV air irradiation on viral diseases transmitted via the respiratory tract [64,65].

These last three studies do not disprove aerosol transmission. Such transmission could have occurred at lower room levels and the schoolchildren were free to contract infections outside of the classroom. However, one might have expected some decrease in infection rates. Furthermore, their negative results stand in stark contrast to the dramatic effects seen in the irradiated patients in Livermore, leading us conclude the irradiated Livermore patients were the beneficiaries of more than just cleaner air.

## What is the serial interval for influenza?

*The generally accepted theory of sick-to-well transmission demands direct epidemiological measurement (not calculation from the incubation period) of a serial interval between causal and resultant cases (time between successive cases in a chain of transmission) as has been amply demonstrated for other respiratory infectious diseases*. In families, where the virus infects one member outside the home and that member then infects others inside the family, a serial interval should be easy to demonstrate if the virus is propagating itself via sick-to-well transmission. Unfortunately, when the World Health Organization Writing Group reported that "the serial interval ... is 2 – 4 days" (p. 83) for influenza, they failed to give a reference and apparently meant the incubation period is 2 – 4 days [56]. While the incubation period of influenza is well documented, if anyone has successfully documented a serial interval for influenza in families, we have yet to locate their work.

In contrast, Hope-Simpson, using viral isolates obtained over 8 years, found low attack rates within households, a high proportion of affected households with only one influenza case (70%), and no demonstrable serial interval [66]. A five-year serological surveillance study found that 73% of family members who get influenza get it on the first day and are apparent index cases [67]. They could not identify a serial interval. Jordan *et al *followed 60 families during the Asian epidemic of 1957, isolating the virus from 86% of the families [68]. They found no evidence of a serial interval. Jordan later reviewed similar studies and reported, "No peak occurred at the expected incubation period when secondary cases in families were plotted by intervals from the index case" [61]. (p. 32).

Viboud *et al *did not say so, but they apparently could not demonstrate a serial interval in families, as secondary cases did not peak at any particular interval after the first case in the family [69]. Remarkably, in 116 families, two family members developed symptoms simultaneously. Of the 131 family members who developed a flu-like illness within five days of the 543 serologically confirmed first cases, it appears that 38 of 131 occurred on day one, 40 on day two, 30 on day three, 28 on day four, and nine on day five.

If influenza is highly contagious, a serial interval should be evident – easily observed and directly measured – as sick family members infect the well. The large percentage of family members that sicken on the first day and the lack of a demonstrable serial interval, despite numerous attempts to measure one, seems more consistent with a limited number of infectors, usually outside the family, than with all the sick being infectors.

## What is the secondary attack rate for influenza?

The reproductive number, R_0_, an estimation of the average number of new cases of influenza produced by each infectious case in a fully susceptible population, has replaced secondary attack rates in most epidemiological models. However, the R_0 _for influenza has been "notoriously hard to estimate" [70] (p. 11146). While the estimated R_0 _remains obscure, epidemiologists have directly measured its father, secondary attack rates, for more than 5 decades. For a highly infectious virus, secondary attack rates for influenza are surprisingly low.

Secondary attack rates for influenza cannot be accurately determined without knowing the serial interval and are thus actually subsequent attack rates. Subsequent attack rates inflate the rate because they include all co-primary, tertiary, and later cases as secondaries. The subsequent attack rate for rhinovirus among non-immune family members is 58% [71]. The rate for unvaccinated household contacts is 70% for measles [72] and 71% for varicella [73]. If influenza is highly contagious and spread by the sick, then secondary attack rates should reflect that contagiousness.

However, 80% of household members with an infected family member escaped the first outbreak of Hong Kong influenza in Great Britain despite it being a new antigenic variant in a non-immune population [74]. Thus, even if one assumes all subsequent cases were secondaries, the secondary attack rate was only 20%. Neuzil *et al *found that 22% of household members became ill within three days of a child in the family being absent from school due to illness but did not report how many family members became ill on the same day as the child [75]. Using a specific clinical definition in secondary cases, Viboud *et al *found a subsequent attack rate of 18% [69].

Longini *et al *analyzed data from four large family studies, reporting the apparent secondary attack rates varied from 13 to 30% [76]. After taking the community infection rate into account, they concluded the actual secondary attack rate among family members was 15%. Later, Longini *et al *estimated the secondary attack rate for adults and children with low levels of preexisting viral specific antibodies was 18 percent and 37%, respectively, while the secondary attack rate in adults and children with high levels of such antibodies was 1.6% and 3.4%, respectively [77].

For a review of all studies on subsequent attack rates up to 1986, see Thacker [78]. Of the eight household studies he analyzed, four showed a subsequent attack rate in the teens (14%, 15%, 15%, 17%), two in the twenties (21% and 27%), one was 31%, while one was 58% (H_3_N_2 _in New Zealand in 1973). The weighted mean of subsequent attack rates in all 870 households was 22%.

A recent review combining the data from four controlled household studies of antiviral effectiveness in the control households found a combined subsequent attack rate of 13% for symptomatic laboratory confirmed infections (136 of 1061 contacts) and 23% for any laboratory confirmed infections (246 of 1061 contacts) [79].

Such low subsequent attack rates in families seem inconsistent with a highly infectious virus sustaining itself by sick-to-well transmission. They seem more consistent with large intrafamilial variations in immunity and family members contracting the infection, usually outside the home, from a common source.

## Animal studies

Ironically, the strongest evidence for sick-to-well transmission in man comes from studies of ferrets. Unlike human studies, studies show infected ferrets readily transmit influenza to well animals and those newly sickened animals readily infect a third animal and so on [80]. Recently, similar experiments with guinea pigs were able to sustain a chain of eight successive transmissions but the animals do not become ill (written communication with Lowen A., Palese Laboratory). Likewise, hamsters can transmit influenza but apparently do not become ill [81]. Schulman and Kilbourne were able to infect about 50% of secondary mice after caging them with a two experimentally infected animals [82]. However, they were unable to get the newly sickened mice to transmit, that is, instigate a chain of transmission from sick to well mice.

Schulman and Kilbourne did demonstrate that some infector mice are "good transmitters" while other mice will not transmit the virus, it spite of inoculation with the same dose of virus. That is, for unknown reasons, some infected mice readily transmit the disease to their littermates and some will not. As all infector mice received an identical inoculum of virus, it is reasonable to hypothesize that good transmitters have an unidentified inadequacy in innate immunity that facilitate their ability to transmit the virus.

It is worth noting that one animal study indicated vitamin D, when added to the diet of rats, prevented influenza but a subsequent paper reported it did not [83,84]. Young *et al *also reported that a Japanese researcher, Midzuno, was able to reproduce influenza in rats simply by maintaining them on diets deficient in vitamin D, apparently part of Japan's World War II biological weapons research. (The American CIA confiscated Midzuno's papers after the war.) As vitamin D does not upregulate AMPs in murine mammals, it is unclear what these studies mean. If researchers can identify an influenza susceptible species in which vitamin D increases expression of AMPs, it would be useful to know if vitamin D deficiency promotes the pathology of influenza.

## Discussion

After a 20 year search for parsimony, Hope-Simpson hypothesized that influenza is mainly transmitted by a limited number of highly infectious latent carriers – carriers infected the prior season – who are called into infectivity by a "seasonal stimulus" inextricably bound to sunlight and who remain highly infective for brief periods, thus explaining the waves of influenza that abruptly end despite a wealth of non-immune potential victims [2]. Nevertheless, to our knowledge, researchers have never demonstrated latency for influenza, as expected with a constantly replicating RNA virus.

However, significant seasonal and population variations in innate immunity make it unnecessary to postulate latency to explain the bizarre epidemiology of influenza. While any theory of influenza must take into account four factors: transmissibility, virulence, adaptive immunity, and innate immunity, it has been easy to ignore innate immunity as it lacked demonstrable seasonal variations, population variations, and a mechanism of action.

To make sense of influenza's epidemiology, we revise Hope-Simpson theory, hypothesizing marked variation in the infectivity of the infected (the good infectors demonstrated in rats by Schulman and Kilbourne in 1963) and that vitamin D deficiency is Hope-Simpson's seasonal stimulus. Adding these two factors to transmissibility, virulence, and adaptive immunity, solves a number of influenza's mysteries.

1. Why is influenza both seasonal and ubiquitous and where is the virus between epidemics?

If influenza were surviving in an endless chain of transmissions from good transmitters to the well – the good transmitters being generally asymptomatic during times of enhanced innate immunity – the disease would be widely seeded in the population, explaining its ubiquity. Seasonal impairments in innate immunity would allow seasonal epidemics in temperate latitudes and less predictable epidemics in tropical zones, depending on viral novelty, transmissibility, virulence, and the innate immunity of the population. Non-seasonal isolated outbreaks would usually only appear in nursing homes [85] or prisons [86] where lack of sunlight impaired innate immunity; such isolated outbreaks would seldom lead to community outbreaks. More extensive out-of-season outbreaks, as occurred in 1918, would arise when novel antigenic viruses with significantly greater infectivity and virulence overwhelm innate immunity.

2. Why are influenza epidemics so explosive?

Predictable fall and winter impairments in innate immunity in temperate latitudes – and less predictable recurrent impairments in subequatorial and equatorial latitudes – would cause a percentage of the non-immune population to become suddenly susceptible to background influenza virus. The size of that susceptible subpopulation would vary, not only by the size of their impairments in innate immunity, but with the transmissibility and virulence of the virus, and the percentage of the population with competent adaptive immunity. Abrupt deficiencies in innate immunity, especially when large segments of the population also have inadequate adaptive immunity, would allow quiescent influenza to erupt.

3. Why do epidemics end so abruptly?

The rapid depletion of the population with both impaired innate and inadequate adaptive immunity may explain the abrupt disappearance of influenza. Impairments in innate immunity may also increase transmission, in effect, turning more infectors, symptomatic or not, into good transmitters. Furthermore, if only a small population of good transmitters – and not all the sick – usually spread the virus, and their transmission period is limited, the epidemic would end shortly after the good transmitters lose their infectivity.

4. What explains the frequent coincidental timing of epidemics in countries of similar latitudes?

Simultaneous impairments of innate immunity at similar latitudes – due to seasonal sunlight deprivation – explain the almost simultaneous eruption of influenza at sites of different longitude but similar latitude. If the virus had already imbedded itself in a population and a subgroup of the infected became good transmitters when their innate immunity declines to a critical threshold, such transmitters would coincidentally infect populations at similar latitudes made susceptible by those same impairments in innate immunity.

5. Why is the serial interval obscure?

Good transmitters explain the difficulty identifying influenza's serial interval especially since influenza's incubation period is well known. If only subpopulations of infected persons are good transmitters, and if their infectious period is limited, then the serial interval would remain obscure until we identified the good transmitters. Vitamin D induced variations in natural immunity may also affect influenza's incubation period, further obfuscating the serial interval.

6. Why is the secondary attack rate so low?

The studies we identified found a secondary attack rate of around 20%, impossibly low for a highly infectious virus spread from the sick to the well. If only a subpopulation of the infected, the good transmitters, are infective, this would explain the surprisingly low secondary attack rates. Current estimates of secondary attack rates assume the first case in the family is the index case and is spreading the disease. However, if only a subpopulation of infected persons transmit the disease, the true secondary attack rate could not be accurately determined until we identify the good infectors.

7. Why did epidemics in previous ages spread so rapidly, despite the lack of modern transport?

If influenza were embedded in the population, only to erupt when impairments in innate immunity create a susceptible subpopulation, the disease would only give the appearance of spreading. Instead, it would appear in large segments of the population seasonally, and almost simultaneously, as long as good transmitters were available. Furthermore, as good transmitters traveled, populations with neither adequate innate immunity nor competent adaptive immunity may succumb. That is, the disease would actually spread, as good transmitters traveled and subsequently infected well subpopulations with impaired immunity.

8. Why does experimental inoculation of seronegative humans fail to cause consistent illness?

If influenza is highly infectious, one would expect most, if not all, human volunteers iatrogenically inoculated with a novel virus to fall ill. Although the rate of illness depends on the virus used and the dose of the inoculum, variations in the innate immunity of the volunteers also explain such variable illness response. We propose individual variations in 25(OH)D levels explain some degree of the variations in illness response.

9. Over the last 20 years, why has influenza mortality in the aged not declined with increasing vaccination rates?

Given that influenza vaccines effectively improve adaptive immunity, the most likely explanation is that the innate immunity of the aged declined over the last 20 years due to medical and governmental warnings to avoid the sun. While the young usually ignore such advice, the elderly often follow it [87,88]. We suggest that improvements in adaptive immunity from increased vaccination of the aged are inadequate to compensate for declines in innate immunity the aged suffered over that same time.

## Conclusion

Kilbourne once wrote the "student of influenza is constantly looking back over his shoulder and asking 'what happened?' in the hope that understanding of past events will alert him to the catastrophes of the future" [89]. That is all we are attempting.

Certainly, without factoring in the effects of innate immunity, we must contort our logic to make sense of influenza's bewildering epidemiological contradictions. When seasonal and population variations in innate immunity are considered in context with the novelty, transmissibility, and virulence of the attacking virus, the conundrums are fewer. A subpopulation of good transmitters among the infected further clarifies influenza's confusing epidemiology. The addition of both variables would improve current epidemiological models of influenza.

Compelling epidemiological evidence indicates vitamin D deficiency is the "seasonal stimulus" [22]. Furthermore, recent evidence confirms that lower respiratory tract infections are more frequent, sometimes dramatically so, in those with low 25(OH)D levels [90-92]. Very recently, articles in mainstream medical journals have emphasized the compelling reasons to promptly diagnose and adequately treat vitamin D deficiency, deficiencies that may be the rule, rather than the exception, at least during flu season [40,41]. Regardless of vitamin D's effects on innate immunity, activated vitamin D is a pluripotent pleiotropic seco-steroid with as many mechanisms of action as the 1,000 human genes it regulates [93]. Evidence continues to accumulate of vitamin D's involvement in a breathtaking array of human disease and death. [40,41]

In 1992, Hope-Simpson predicted that, "understanding the mechanism (of the seasonal stimulus) may be of critical value in designing prophylaxis against the disease." Twenty-five years later, Aloia and Li-Ng found 2,000 IU of vitamin D per day abolished the seasonality of influenza and dramatically reduced its self-reported incidence [25]. (Figure 2) Hence, we propose this modification of Hope-Simpson's theory. We do not expect our revisions to prove invincible, nor do we delude ourselves that influenza is now comprehensible. Rather, we build on Hope-Simpson's theory so that it "may be corroborated, corrected, or disproved." (Hope-Simpson, 1992, p. 191)

## Abbreviations

AMPs: antimicrobial peptides; RCT: randomized controlled trial; Pathogen Associated Molecular Patterns: PAMPS

## Competing interests

Dr. Cannell heads the non-profit educational group, 'The Vitamin D Council'.

## Authors' contributions

JJC conceived of the project, consulted with EG, and wrote each new draft. MZ added material on innate immunity. CFG and RS revised the first and subsequent drafts and expanded the article's scope. EG revised and reviewed all drafts and added additional material to each draft. All authors read and approved the final manuscript.
